# Hydroxycinnamic Acids and Carotenoids of Dried Loquat Fruit cv. ‘Algar’ Affected by Freeze-, Convective-, Vacuum-Microwave- and Combined-Drying Methods

**DOI:** 10.3390/molecules25163643

**Published:** 2020-08-10

**Authors:** David Bernardo López-Lluch, Marina Cano-Lamadrid, Francisca Hernández, Aleksandra Zimmer, Krzysztof Lech, Adam Figiel, Ángel Antonio Carbonell-Barrachina, Aneta Wojdyło

**Affiliations:** 1 Departamento Economía Agroambiental, Escuela Politécnica Superior de Orihuela (EPSO), Universidad Miguel Hernández de Elche (UMH), Carretera de Beniel, km 3.2, 03312 Orihuela, Alicante, Spain; david.lopez@umh.es; 2 Departamento Tecnología Agroalimentaria, Grupo Calidad y Seguridad Alimentaria, Escuela Politécnica Superior de Orihuela (EPSO), Universidad Miguel Hernández de Elche (UMH), Carretera de Beniel, km 3.2, 03312 Orihuela, Alicante, Spain; marina.cano.umh@gmail.com (M.C.-L.); angel.carbonell@umh.es (Á.A.C.-B.); 3 Department of Plant Sciences and Microbiology, Group Plant Production and Technology, Escuela Politécnica Superior de Orihuela (EPSO), Universidad Miguel Hernández de Elche (UMH), Carretera de Beniel, km 3.2, 03312 Orihuela, Alicante, Spain; francisca.hernandez@umh.es; 4 Institute of Agricultural Engineering, Wrocław University of Environmental and Life Sciences, 37a Chełmońskiego Street, 51-630 Wrocław, Poland; zimmer.ola@gmail.com (A.Z.); krzysztof.lech@upwr.edu.pl (K.L.); adam.figiel@upwr.edu.pl (A.F.); 5 Department of Fruit, Vegetable and Plant Nutraceutical Technology, Wrocław University of Environmental and Life Sciences, 37 Chełmońskiego Street, 51-630 Wrocław, Poland

**Keywords:** *Eriobotrya japonica* Lindl, convective, vacuum-microwave, bioactive compounds, antioxidant capacity

## Abstract

The effect of different drying techniques (freeze, convective, vacuum-microwave and combined drying) on the drying kinetics, the phytochemical compounds and sensory characteristics in loquat cultivar ‘Algar’ was studied. The convective drying resulted in the highest amount of total hydroxycinnamic acids (5077 mg/kg wet weight (ww)), with 3-caffeoyl quinic acid and 5-caffeoyl quinic acid being the greatest carotenoids. The highest values of total carotenoids were obtained by the freeze-drying technique (2601 mg/kg ww), followed by all convective treatments and vacuum-microwave at 360 W. The highest carotenoid was β-carotene. The ABTS^+•^ (2,2′-azino-bis(3-ethylbenzothiazoline-6-sulfonic acid)) and FRAP (Ferric Ion Reducing Antioxidant Power) values ranged from 2.04 up to 3.27 mmol Trolox/100 g ww, and from 1.89 up to 2.29 mmol Trolox/100 g ww, respectively. As expected, the color difference of freeze-dried samples was the lowest (7.06), similar to combined drying conditions (9.63), whilst the highest value was found after convective drying (37.0). All treatments were sensory acceptable (no off-flavors). However, still, further research is needed to fully optimize these studied drying treatments because the freeze-dried sample still had higher carotenoid content and better instrumental color parameters. Although recently the impact of microwave drying has been studied, this is the first work comparing phytochemical composition of loquat fruit under the different drying methods mentioned above.

## 1. Introduction

The loquat species (*Eriobotrya japonica* Lindl.) comes from China and it was introduced into Europe in the XVIII century, but only as an ornamental tree in Botanical Gardens. In Spain, it was introduced two hundred years ago, and it was already used for productive ends, as well as for ornamental [1]. Although there are no FAO (The Food and Agriculture Organization) statistics available for world loquat fruit production, Lin [2] reported 550,000 t for 2006 [2], with China being the main producing country followed by the Mediterranean basin, especially Spain [3]. Spain is the world’s major exporter, and more than 50% of Spanish loquat production (28,522 t in 2017) is cultivated in the southeastern region of the country. The most important Spanish production area is Alicante (14,160 t in 2017), followed by Granada (10,716 t in 2017) [4]. The most popular are ‘Nísperos Callosa d’En Sarriá’, for which production has been safeguarded by a Protected Designation of Origin (DOP) since 1992 (R (UE) 1992/2081). Among loquat cultivars with the above-mentioned DOP (‘Algar’, ‘Nadal’, ‘Golden’ and ‘Magda’), not only is the ‘Algar’ cultivar the predominant one (98% of the total production) [5], it is also the most recognized one due to its specific yellowish peel and flesh, sweetness and slight sourness, and rounded size [6]. These reviewed characteristics correspond to the description of the product, which relates them to their local environment, and conditions of cultivation and processing (located in family orchards, in high-density plantations, using drip irrigation).

Phenolic compounds are secondary metabolites of plants and contribute to color, basic taste and flavor. Focusing on no-flavonoid compounds, the highest values have been found in peel and leaves due to the UV (ultraviolet) filters’ activity [7] and is related to healthy properties such as anticancer, chronic and cardiovascular disease [8]. Previous studies have indicated that loquat peels, leaves and flesh are rich in 5-caffeoylquinic and 5-feruloylquinic acids: 3-caffeoylquinic, 3-*p*-coumaroylquinic, 5-caffeoylquinic and 5-feruloylquinic acids, and 5-caffeoylquinic acid and 3-caffeoylquinic, respectively [9].

Regarding carotenoids, one of the most known healthy benefits is against eye disease [10]. Recent studies evidenced that not only were differences between flesh and peel carotenoid profiles important, but also those between the cultivar and stage of maturity. The most observed common compounds have been *trans*-lutein, *trans*-β-carotene and *trans*-β-cryptoxanthin, and *trans*-β-carotene and 5,8-epoxy-β-carotene in the peel and flesh, respectively [11,12,13].

The incidence of post-harvest disorders as a chilling injury (external and internal browning) and decay by ethylene action are the main problems for this fruit during storage [13,14]. Products based on dried loquat seem to be a great opportunity to prolong loquat consumption, promote this crop and to avoid the lack of knowledge of the consumers about their health benefits, as other authors have recently reported [15]. Currently, in low production, jams, beverages, syrup and dried loquats are available on the market. The sensory attributes of some of these products are mostly not acceptable for consumers, especially dried loquat due to intense browning. On the other hand, due to the shortened times by using temperature-controlled microwave heating with infrared thermography, loquat fruits cv. ‘Peluche’, ‘Virticchiara’ and ‘Claudia’ guaranteed high quality, both from a functional and sensory point of view [15].

As far as we are aware, no research involving the effect of convective drying (CD), vacuum-microwave drying (VMD) and combined drying (CD-VMD) methods on the drying kinetics, the phytochemical compounds and sensory characteristics in loquat cultivar ‘Algar’ has been published. This study aimed to describe the impact of the amount of hydroxycinnamic acids and carotenoids of dried loquat by the above-mentioned methods and assess the antioxidant potential of the above-mentioned treatments. To achieve these aims, antioxidant compounds were determined by LC-PDA/MS (liquid chromatography with a photodiode array coupled directly with Mass Spectrometry) and antioxidant properties were checked against ABTS^+•^ and FRAP assay.

## 2. Results and Discussion

### 2.1. Drying Kinetics

**Figure 1:**
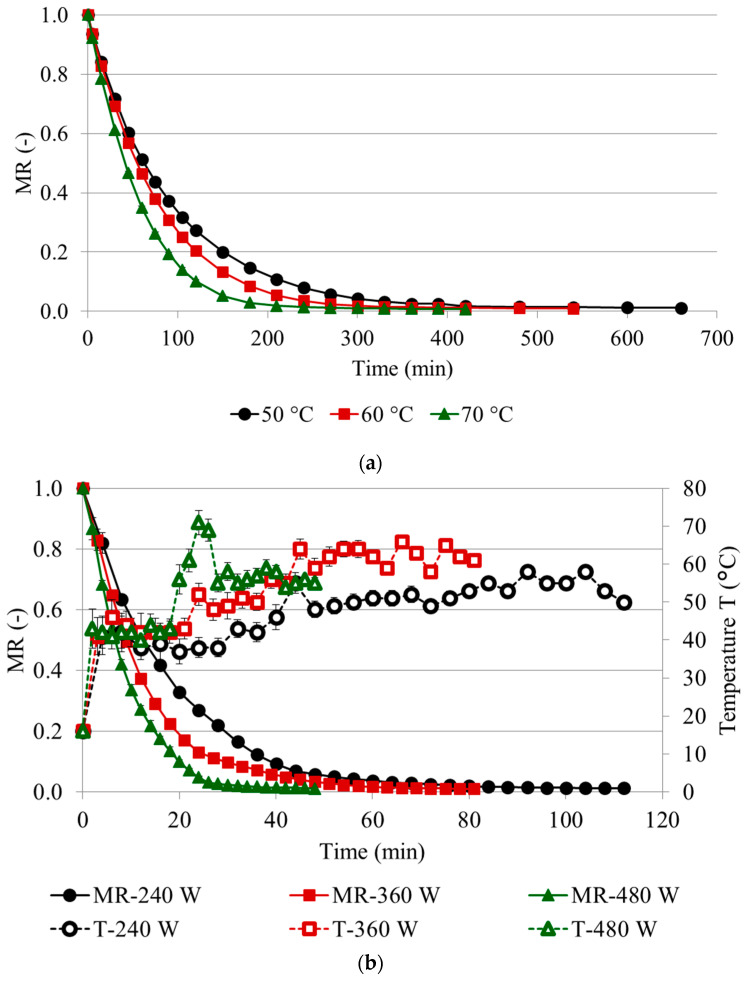

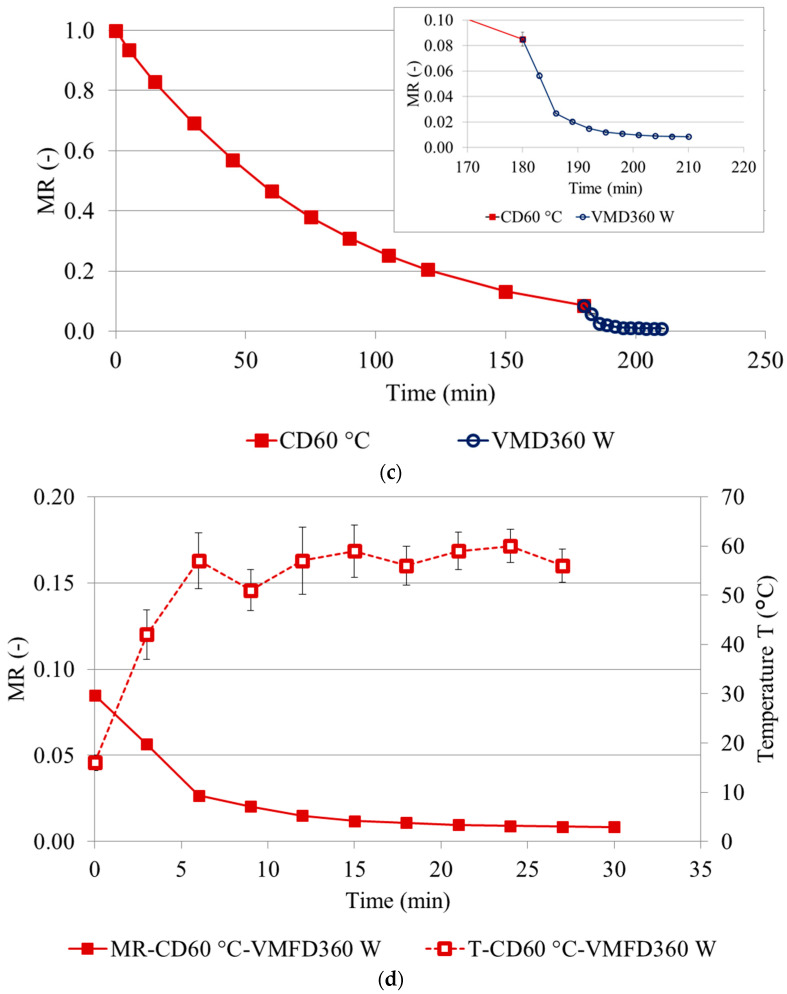
Drying kinetics of loquat fruit dehydrated by the studied drying conditions: (**a**) CD at 50, 60 and 70 °C, (**b**) VMD at 240, 360 and 480 W, (**c**) CPD at 60 °C followed by VMFD (360 W) with emphasis on VMFD (**d**). MR: moisture ratio. Convective drying (CD) at 50 °C (CD 50), 60 °C (CD 60) and 70 °C (CD 70), vacuum-microwave drying (VMD) at 240 W (VMD 240), 360 W (VMD 360) and 480 W (VMD 480) and combined drying (CPD-VMFD).

The drying kinetics represents a decreasing moisture ratio (MR) at the time of drying. Preliminary tests conducted in this study proved that the best fitting was obtained for the modified Page model (Table 1), and only this model was used in this study: where A, n and k are constants. The changes in the moisture ratio (MR) of loquat samples dehydrated by CD at 50, 60 or 70 °C, VMD at 240, 360 and 480 W and the combined CPD-VMFD are presented in Figure 1. The modified Page model, one of the models derived from Newton’s law of cooling, was found to be the best model for loquat fruits during CD, VMD and VMFD as a final part of combined drying. It has already been successfully used to describe the drying kinetics of different fruits such as banana, kiwifruit, mango, persimmon, quince and pomegranate [16]. It is worth mentioning that there are no previous data about drying kinetics of loquat fruit. In the current study, the root mean square error (RMSE) ranged between 0.0046 and 0.0142, with the coefficient of determination (*R*^2^) being between 0.9595 and 0.9998 (Table 1). These values (high *R*^2^ and low MSE) proved the good agreement between the thin layer modelling equation and the experimental data [16]. In addition, basic information concerning the influence of different drying conditions on drying time and final moisture content of dried samples is shown in Table 1. The final moisture content of dried products (MC) was in the range from 6.39 to 8.38 g/100 g ww, depending on the drying conditions applied. Both this low moisture content and water activity (<0.400) guaranteed the microbiological safety of the dried product. Results were in agreement with a previous study which indicated that MC < 10 g/100 g ww is microbiologically safe [17].

In the initial stage of CD, a fast loss of water was observed, regardless of the drying temperature applied (Figure 1a). The second stage caused a reduction in the intensity of water removal from loquat samples due to a significantly lower drying rate. The reason is that CD efficiently removed the surface moisture by using surface heating, but the removal of internal moisture was limited [18]. Increasing the temperature of hot air from 50 to 70 °C decreased the drying time from 660 to 420 min (36%). Recently, a 63% and 40% reduction of drying time was noticed during CD of loquat flowers from 40 to 80 °C and from 50 to 80 °C, respectively [19]. A similar trend was also found in the case of quince slices, where increasing the temperature of hot air from 50 to 70 °C decreased the drying time from 300 to 240 min (20%) [17]. That decrease in the drying time agreed with the increase of the parameter n of the Page model, from 0.96 to 1.08. Figure 1b shows the drying kinetics of loquat fruit dehydrated by VMD; although similar water removal behavior was found during CD, a higher drying rate during the initial period of VMD was observed, as in previous studies [18]. Increasing the microwave power from 240 to 480 W decreased the drying time from 112 to 48 min (57%), which was associated with the increased values of both parameters *k* and *n* from 0.053 to 0.079 and from 1.02 to 1.13, respectively. A similar trend was found in marjoram (50%) [20] and strawberries (51%) [21]. Although VMD is a great deal faster than CD, which was confirmed by much higher values of the *k* parameter, it is not recommended as a single application due to the high investment and operating costs resulting from relatively large vacuum installation and electrical supply. Convective pre-drying (CPD) has been indicated as more effective at the first stage of drying [22]. CPD was followed by the vacuum-microwave finish drying (VMFD) at the range of MR indicated by the value of parameter *A,* amounted to 0.086. Combined-drying, compared to CD at 60 °C, considerably reduced the drying time (≈60%) (Figure 1c,d).

The temperature reached during drying is one of the most important parameters due to the impact on the amounts of bioactive compounds in the final product. Not only does the temperature depend on microwave wattage/power applied [23], but the balance of the energy generated by water molecules inside the material under microwave radiation and the energy necessary for water evaporation should also be taken into account [17].

During the increase in the microwave power, from 240 to 360 W and to 480 W, the maximum temperatures reached for dried loquat were from 52 to 64 °C and to 71 °C, respectively (Figure 1b). As for the combined drying, CPD-VMFD, the temperature reached was 60 °C during the entire 210 min of drying, including 30 min of VMFD (Figure 1d).

### 2.2. Hydroxycinnamic Acid Derivatives

Table 2 and Table 3 show identified and quantified individual hydroxycinnamic acids (H; *n* = 6) in dried loquat, respectively: (*i*) 3-caffeoylquinic acid (3-CQA), (*ii*) 3-*p*-coumaroylquinic acid (3-*p*-CoQA), (*iii*) 5-caffeoylquinic acid (5-CAQ), (*iv*) sinapoyl glucoside (SG), (*v*) 5-*p*-coumaroylquinic acid (5-*p*-CoQA) and (*vi*) 5-feruloylquinic acid (5-FQA). The hydroxycinnamic acid profile was predominated by 5-CAQ (mean of all samples = 2610 mg/kg ww), followed by 3-CQA (1289 mg/kg ww) > 3-*p*-CoQA (272 mg/kg ww) > 5-CAQ (155 mg/kg ww) ≈ 5-FQA (160 mg/kg ww) > 5-*p*-CoQA (16.2 mg/kg ww). Our results were in agreement with a previous study which identified these compounds and a similar profile in loquat flesh [9]. No previous study has researched the effect of drying techniques on hydroxycinnamic acid of loquat fruit.

The amount of total hydroxycinnamic acid derivatives (∑H), especially 5-CAQ, presents an important role in manufacturing into processed products, such as drying, since they are part of the catecholase activity of the polyphenol oxidase as a substrate [17,18,19,20,21,22,23,24]. Therefore, drying processes influenced both the amount of individual hydroxycinnamic acid and ∑H in dried loquat. Focusing on the major compounds (≈90% of ∑H was 3-CQA and 5-CAQ), there were no significant differences among CD 50 °C, CD 60 °C and CD 70 °C, maintaining the highest amount of these two compounds, and consequently, the highest ∑H (mean of the mentioned treatments, 5077 mg/kg ww). The sum of hydroxycinnamic acids in CD samples was higher by 10% when compared to FD, making this method competitive to dehydration, being in accordance with a previous study of dried plum powders [25]. It is worth mentioning that it was slightly noticed that the higher the temperature, the higher the amount of hydroxycinnamic acids.

The greatest degradation of both these compounds and the sum of hydroxycinnamic acids was found in VMD 240 (25% less compared to FD) by reducing mainly 5-CAQ, followed by 3-CQA. Among FD, VMD 360 and VMD 480, no statistical differences were observed. It is necessary to highlight that the values of CPD-VMFD (maximum 60 °C) maintained statistically higher than VMD methods and FD, whilst the amount was significantly lower than CD methods. A large percentage of phenolic compounds are bound to cellular structures and mild drying treatments release bound phytochemicals from the matrix, being more accessible.

### 2.3. Carotenoids

Table 2 and Table 3 show identified and quantified individual carotenoids (C; *n* = 7) in dried loquat, respectively: (*i*) Zea: zeaxanthin, (*ii*) β-C: β-carotene, (*iii*) *cis*-β-C: 15,15-*cis*-β-carotene, (iv) β-Cp: ß-cryptoxanthin-palmitate, (*v*) Neox: neoxanthin, (*vi*) Zeam: zeaxanthin monopalmitate and (*vii*) β-Cpm: β-cryptoxanthin monopalmitate. The carotenoids profile was predominated by β-carotene (mean of all samples = 571 mg/kg ww), followed by β-cryptoxanthin–palmitate (222 mg/kg ww) ≈ β-cryptoxanthin monopalmitate (222 mg/kg ww) > zeaxanthin monopalmitate (101 mg/kg ww) > neoxanthin (99.0 mg/kg ww) > 15,15-*cis*-β-carotene (62.7 mg/kg ww) > zeaxanthin (29.3 mg/kg ww). Our results were partially in agreement with a recent study which observed that the predominant carotenoid in the ‘Algar’ loquat pulp was 15-Z-phytoene, followed by β-carotene and β-Cryptoxanthin–palmitate, neoxanthin, zeaxanthin monopalmitate and β-cryptoxanthin monopalmitate [13]. Recently, the major carotenoid of dried loquat cv. ‘Virticchiara’ and ‘Claudia’ was found to be all-*trans*-βcarotene, while β-cryptoxanthin was predominant in dried loquat cv. Peluche [15]. As it is well-known that carotenoids are destroyed by heat treatment, a decrease of the amounts of total carotenoids is expected, as previous studies indicated a loss between 6% and 19% [26] and a loss between 24% and 42% [15]. Zeaxanthin (Zea) was the most stable carotenoid, with no significant differences among studied drying techniques, whilst it was observed by other authors that β-carotene was the most stable during the goji drying treatment [27]. On the other hand, Farina et al. [15] indicated a 51% loss of zeaxanthin after microwave drying at 60 °C. What is more, zeaxanthin dipalmitate losses (31–41%) were higher than those in a previous study (15–20%) observed in goji berries [27].

According partially to other authors [28], the retention is significantly improved by reducing the processing time (VMD against CD, and combined method against CD) and lowering the temperature (CD 50 °C against CD 70 °C). Contrary to our results, other authors indicated that an alternative to increase the retention of carotenoids is a reduction of levels of oxygen in the absence of light, such as microwave and vacuum drying methods [29].

Due to the fact that all-*trans*-β-carotene is very unstable, it can be easily isomerized into cis-isomers, when exposed to heat and light [30]. Previous studies about carotenoids concluded that there is an increase in cis isomers of β-carotene during thermal processes [31,32], being in agreement to our results. Farina et al. [15] observed a loss of about 43% and 71% of 9-*cis*-β-carotene and 13-*cis*-β-carotene after microwave drying of loquat fruit. Isomerization energy is involved in relocation of the single or double bond of one form of carotenoid into another.

### 2.4. Antioxidant Capacity

As shown in Table 3, the antioxidant capacity was affected by the drying system. The ABTS^+•^ and FRAP levels from dried loquat were increased with the increase of CD temperature from 50 to 70 °C. A similar effect was obtained for loquat fruit [33], which obtained the higher temperature vacuum drying (70 to 140 °C) and the higher antioxidant levels in loquat fruit. However, our results disagree with those obtained for apple cubes [34], pomegranate arils [35], dried jujube [36] and dehydrated pomegranate [37], which concluded that antioxidant capacity, measured by DPPH, ABTS^+•^ and FRAP, values decrease as the drying temperature increases from CD 50 to CD 70 °C. Therefore, the increase observed of our samples compared with other fruits could be due to the difference of composition and the formation of compounds with antioxidant capacity. On the other hand, the ABTS^+•^ and FRAP values for vacuum-microwave drying (VMD) dried loquat were also increased with the power levels. A similar trend was described for pomegranate arils [35] and jujube fruits [36], which obtained the higher values of ABTS^+•^ and FRAP for vacuum-microwave drying at 480 W, however, other authors [37] obtained lower values of antioxidant capacity, measured by DPPH, in pomegranate at VMD 480 W. Regarding the CPD-VMFD, it showed the lowest values of ABTS^+•^ and FRAP (2.23−2.29 mmol Trolox/100 g ww, respectively). Finally, for the values of antioxidant capacity, measured by ABTS^+•^ and FRAP, CD-dried loquat presented the best results at 70 °C, followed by the vacuum-microwave drying at 480 W (Table 3), whilst the lowest antioxidant capacity values were obtained by CPD-VMFD and FD. One reason why the antioxidant capacity of dried loquats is greater as the drying temperature increases may be due to the fact that a part of polyphenols and flavonoids were transformed from combined state to free state at high temperature. Choi et al. [38] reported that the overall free polyphenolic and flavonoid compounds of Shiitake were increased by heat treatment, while the total bound polyphenols and flavonoids declined with increasing both heating time and heating temperature.

### 2.5. Color Coordinates

The drying method significantly (*p* < 0.001) affected lightness (L*), green-red coordinate (a*) and blue-yellow coordinate (b*) in loquat samples (Table 1). Among the samples analyzed, the darkest values were found in the samples VMD 240W and VMD 360 W, followed by VMD 480 W and CD 70 °C, and their mean values were 1.5–1.6 lower than those of FD samples. The application of drying techniques resulted in a non-significant decrease in a* values, as compared to samples obtained using FD. The level of yellow pigments, as described by the coordinate b*, was affected by drying method. The highest b* values were found in the samples: CD 60 °C (18.5), CD 50 °C (15.7) and CPD-VMFD (15.7), the b* values of these treatments were statistically equivalent to those of the control sample (FD (20.9)). Taking the above-mentioned values into account, hydroxycinnamic acid derivates, as a part of the influence of the oxidation processes, are able to influence the color during the processing of loquat-based products. Pearson’s correlation coefficient showed that the ∑H was positively correlated with the b* coordinate (*R*^2^ = 0.81). Additionally, the b* coordinate was positively linked with 3-CQA and 5-CAQ (*R*^2^ = 0.77 and 0.83, respectively) and inversely correlated with 5-*p*-CoQA (*R*^2^ = −0.96). It can be concluded that hydroxycinnamic acid derivatives play an important role in the final color of the dried loquat fruit, specifically the yellowish color.

It is well known that the carotenoids are correlated with color. Pearson’s correlation coefficient showed that the L* coordinate was positively correlated with β-carotene (*R*^2^ = 0.88), β-cryptoxanthin–palmitate (*R*^2^ = 0.89), neoxanthin (*R*^2^ = 0.83), zeaxanthin monopalmitate (*R*^2^ = 0.93), β-cryptoxanthin monopalmitate (R^2^ = 0.90) and ∑C (*R*^2^ = 0.90), while a* and b* coordinates were positively linked with C1 (0.72 and 0.92, respectively). It can be concluded that carotenoids play an important role in the final color of the dried loquat fruits. Low ΔE values represent high similarity to the ideal color of fresh vegetal material, and a good performance of the drying method [39]. The color difference (ΔE) of samples ranged from 7.1 to 19.8. The lowest ΔE values were found in the control samples (FD, 7.1), followed by the CPD-VMFD sample (9.7) and by mean value of VMD samples (17.8). It can be stated that application of vacuum-microwave drying resulted in the worst color of the dried loquat.

### 2.6. Descriptive Sensory Analysis

**Figure 2:**
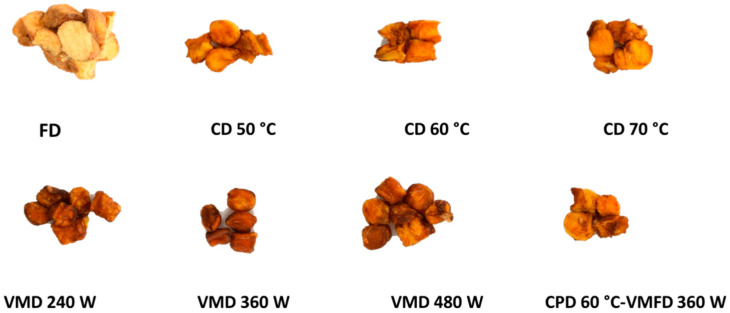
Dried ‘Algar’ loquat by freeze drying (FD), convective drying (CD) at 50 °C (CD 50 °C), 60 °C (CD 60 °C) and 70 °C (CD 70 °C), vacuum-microwave drying (VMD) at 240 W (VMD 240 W), 360 W (VMD 360 W) and 480 W (VMD 480 W) and combined drying (convective pre-drying followed by vacuum-microwave finish drying, CPD 60 °C-VMFD 360 W).

**Figure 3:**
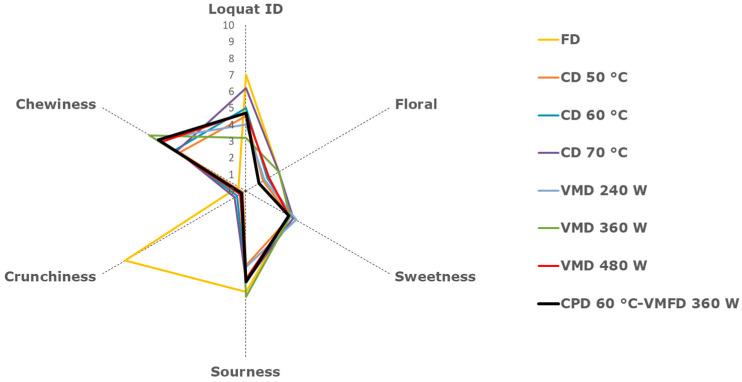
Descriptive sensory analysis of dried loquat. Freeze drying (FD), convective drying (CD) at 50 °C (CD 50), 60 °C (CD 60) and 70 °C (CD 70), vacuum-microwave drying (VMD) at 240 W (VMD 240), 360 W (VMD 360) and 480 W (VMD 480) and combined drying (CPD-VMFD).

The appearance of the samples of the last two treatments can be seen in Figure 2. The main objective of this study was to optimize the loquat flavor of the samples. The values of loquat ID (characteristic sensory basic taste, aroma and flavor of loquat fruit) obtained by the trained panel were: 7.0 (FD) > 6.2 (CD 70 °C) > 5.0 (CD 60 °C) > 4.7 (CPD-VMFD) ≈ 4.7 (VMD 480 W) ≈ 4.5 (CD 50 °C) > 4.0 (VMD 480 W) > 3.2 (VMD 360 W) (Figure 3). It is worth mentioning that none of the samples presented measurable off-flavor notes, being essential for a high quality. Previously, other dried loquat cultivars were analyzed using descriptive sensory analysis, concluding that some unpleasant smell was generated after microwave drying. In addition, this study observed that the typical smell of loquat fruits was slightly lower than that of fresh fruits, while a pleasant odor of honey and caramel was not considered negative by a trained panel [15].

As for the other attributes of the control samples (FD), the predominant one was sourness (6.1), followed by sweetness (3.1) and floral notes (2.3). Sourness changed significantly when CD, VMD and combined drying were used (especially a reduction in CD 50 °C and VMD 240 W), whilst no effect was noticed on sweetness. Regarding floral notes, significant differences were observed, the values were: 2.3 (VMD 360 W) ≈ 2.3 (CD 70 °C) > 1.6 (VMD 480 W) > 1.5 (CD 60 °C) > 1.3 (VMD 240 W) > 1.2 (CD 50 °C) > 0.9 (CPD-VMFD). Besides, three texture attributes were evaluated: hardness, crunchiness and chewiness. It is worth mentioning the high value of crunchiness in FD reaching a value of 8.4, while chewiness value was 0.5. As for crunchiness, a 22-fold, 26-fold and 28-fold decrease was observed in CD, VMD and combined samples, respectively. The effect of CD, VMD and combined drying steps was a 10-fold, 13-fold and 12-fold increase on the chewiness of the dried loquat.

## 3. Materials and Methods

### 3.1. Plant Material and Sample Preparation

Loquat fruits cultivar ‘Algar’ were cultivated in a farm located in Callosa d’En Sarrià, Alicante (Spain), with Protected Designation of Origin (PDO). Loquat fruits (~20 kg) were hand-harvested in mid-April 2019 at a commercial maturity stage (7.5–8.0 °Brix), and immediately posted to Wrocław University of Environmental and Life Sciences (Wrocław, Poland) using the accurate wood-boxes to avoid mechanical damages. The transport took 5 days, stored at 5 °C at an approximate relative humidity (RH) of 90%, which are the optimal storage conditions.

### 3.2. Drying Methodology

Before drying, 20 kg of fresh loquat fruits were cut in cylinders of approximately 8 mm diameter and 8 mm height using a cylindrical punch. Four dehydration technologies were used:

- Freeze drying (FD) was carried out in a freeze-dryer Christ Alpha 1-4 LSC (Martin Christ GmbH; Osterode am Harz, Germany) at a reduced pressure of 65 Pa for 24 h. The temperature within the drying chamber was −60 °C, while the heating plate was at ~30 °C. The freeze-dried loquats were considered as the control sample because it assures the expected quality, as a previous study indicated [39].
- The process of convective drying (CD) was conducted using a drier designed and built at the Institute of Agriculture Engineering (Wrocław, Poland). Loquat samples of around 60 g were spread according to a methodology previously developed [39]. During CD, loquat samples represented by cylindrical particles were spread in a single layer over the mesh of a basket with 100 mm diameter. Drying in a single layer is required when determining the drying kinetics (references). A heavier sample would not form a single layer in the basket. The CD was operated at 3 temperatures: 50, 60 and 70 °C of the drying air flowing with the velocity 0.8 m s^−1^.
- The process of vacuum-microwave drying (VMD) of fresh samples was conducted according to a methodology previously developed [39]. The dryer was operated at 3 power levels: 240, 360 and 480 W, which corresponded to the specific microwave power stream values of 4, 6 and 8 W/g, respectively. These values were used in previous studies on drying other raw materials (references) and thus make possible relevant comparisons. Samples (~60 g) were placed in a cylindrical container of organic glass with a volume of 6.8 L, and with a pressure ranging from 4 to 6 kPa.
- The process of combined drying (CPD-VMFD) consisted of convective pre-drying (CPD) at 60 °C followed by vacuum-microwave finish drying (VMFD) at 360 W.

During VMD, the temperature of dried loquat was measured using an infrared camera i50 (Flir Systems AB, Stockholm, Sweden), immediately after removing the material being dried from the VM drier, and the maximum temperature was recorded. The drying process was carried out until a constant mass of the sample was obtained, which was associated with achieving the lowest possible moisture content (MC) of the sample for the particular drying conditions applied. This approach allowed us to determine the drying kinetics in a wide range of MC values and was conducive to obtaining a crispy texture of the dried product that is desired by consumers and facilitates the milling process to obtain a powdered form of the product. From this perspective, it was possible to assess the suitability of the drying conditions for application purposes limited by the final MC values.

The values of MC were determined as reported previously [40] and expressed as dried basis (db) described by the ratio of the weight of water to the weight of the wet weight (ww). Determination of MC enabled calculation of moisture ratio (MR) by dividing the current value of MC decreasing during drying by the initial value of MC. The course of MR versus drying time represented the drying kinetics of loquat fruits. Modelling of the drying kinetics was performed during preliminary studies. Most typical drying models such as the Newton model, Page model, Henderson and Pabis model, as well as the two-term model were fitted to experimental points representing a decrease of moisture ratio at time of drying. The good fit of a specific model to the experimental data was evaluated using: (*i*) coefficient of determination (*R*^2^) and (*ii*) root mean square error (RMSE). The model fit is accurate if the value of *R*^2^ is closer to 1.0 and the RMSE value is closer to 0.

### 3.3. Identification and Quantification of Hydroxycinnamic acids and Carotenoids

The extraction and determination of hydroxycinnamic acids were performed as described previously by Wojdyło et al. [40]. Grounded dried loquat fruits (~1 g) were mixed with 5 mL methanol/water/ascorbic acid (80:20:1, *v*/*v*/*v*) and sonicated for 15 min (Sonic 6D; Polsonic, Warsaw, Poland). For identification and quantification, 5 μL of each sample was analyzed in a BEH C18 column (2.1 × 10 mm, 1.7 μm; Waters Corp; Dublin, Ireland) at 30 °C with gradient elution at a flow rate of 0.42 mL/min within 15 min. The mobile phase was composed of solvent A (2% formic acid) and solvent B (acetonitrile) as 1% to 25% B solvent until 12 min, and then, held constant to wash and re-equilibrate the column. Quantification of hydroxycinnamic acid was achieved by injection of solutions of known concentrations ranging from 0.05 to 0.5 mg mL^−1^ (*R*^2^ = 0.9998) of chlorogenic acid as a standard. The results were expressed as mg per kg ww (wet weight, after drying technology but not counting MC).

The extraction and determination of carotenoids were performed as described previously by Wojdyło et al. [40]. For identification and quantification, 10 μL of each sample was analyzed in a BEH C18 column (2.1 × 10 mm, 1.7 μm; Waters Corp; Ireland) at 32 °C with gradient elution at a flow rate of 0.5 mL/min within 17 min. The mobile phase was composed of solvent A (0.1% formic acid) and solvent B (acetonitrile:methanol, 7:3, *v*/*v*) as 25% of A until 0.6 min, 4.9% of A until 6.5 min, 0% of A until 13.6 min, and then held constant to wash and re-equilibrate the column. Calibration curves for carotenoids analysis were made from all-*trans*-carotene. The results were expressed as milligrams per kilogram of ww.

The chromatographic conditions for the identification and quantification have been previously reported [36]. The compound identification was done using an Acquity ultraperformance LC (liqud chromatography) system equipped with a photodiode detector (PDA; UPLC) with binary solvent manager (Waters Corp., Milford, MA, USA) series with a mass detector G2 QTof Micro mass spectrometer (Waters, Manchester, UK), equipped with an electrospray ionization (EI) source operating in negative and positive modes.

### 3.4. Trolox Equivalent Antioxidant Capacity (TEAC ABTS^+•^) and Ferric-Reducing Antioxidant Potential (FRAP)

Approximately 1 g of grounded dried loquat in 10 mL of 80% water/methanol/chloride acid (*v*/*v*/*v*) was sonicated for 15 min. After being kept for 24 h at 4 °C in the dark, the extracts were centrifuged (1500× *g* for 10 min at 4 °C). The antioxidant capacity of the extracts was determined using the Trolox Equivalent Antioxidant Capacity test (TEAC ABTS^+•^) according to the method described by Re et al. [41], with slight modifications. The ferric-reducing antioxidant power (FRAP) was analyzed using the method described by Benzie and Strain [42]. Results were presented as mmol Trolox/100 g ww.

### 3.5. Color Measurement and Moisture Content

Color coordinates L*, a* and b* were evaluated using a Minolta Chroma Meter CR-200 Reflectance System (Osaka, Japan). The results, using Illuminant D65 and 10° observer angle, were expressed as L* (lightness), a* (green–red) and b* (blue–yellow) values. These values were then used to calculate hue angle degree (h° = arctang (b*/a*)), where 0° = red–purple, 90° = yellow, 180° = bluish–green and 270° = blue, and chroma (C* = (a*^2^ +b*^2^)^1/2^), indicative of the intensity or color saturation. Color difference (ΔE) was used to describe the difference of the color between dried samples and the fresh control (Equation (1)):

*ΔE* = [(*L* − L*)^2^ + (*a* − a*)^2^ + (*b* − b*)^2^]^0.5^ (1)

The *ΔE* indicates the degree of total color change in comparison to the color of fresh loquat, cv. ‘Algar’ (L* = 47.39, a* = 8.536 and b* = 21.49).

### 3.6. Descriptive Sensory Evaluation

Eight highly trained panelists (>500 h of experience; aged 25 to 50 years; 4 females and 4 males) from the department of Agro-Food Technology (EPSO, UMH, Orihuela, Spain) participated in this study. The panel was selected and trained following the ISO (the International Organization for Standardization) standard 8586-1 (1993), and they are specialized in descriptive sensory evaluation of fruits and vegetables and have a wide expertise in evaluating the sensory quality of different dried fruit and vegetables (e.g., pomegranate, quince, apple, cherry, herbs). For the current study, the panelists received one orientation session of 60 min on fresh and dried loquat. The panel used a numerical scale between 0 and 10, with increments of 0.5, for quantifying the intensity of the loquat dried products’ attributes, where 0 represents no intensity and 10 represents extremely strong intensity. Samples (~4 g of dehydrated loquat) were served in a randomized order and coded using 3-digit numbers. Unsalted crackers and low-mineralization still water were provided to panelists to clean their palates between samples. The panel worked with the lexicon to evaluate only the following attributes: (basic tastes) sweetness and sourness, (flavor) loquat ID and floral and (texture) chewiness and crunchiness. Loquat ID means the characteristic sensory basic taste, aroma and flavor of loquat fruit.

### 3.7. Statistical Analyses

All experiments and analyses were run in triplicate and data reported are presented as the mean of the replications. All data were subjected to the analysis of variance (ANOVA) test and later to Tukey’s multiple range test to determine significant differences among treatments at *p* < 0.05 using XLSTAT Premium 2016 (Addinsoft, NY, USA). Mean values followed by different letters, within the same column, were significantly different (*p* < 0.05). Table Curve 2D (Systat Software, San Jose, CA, USA) was used for fitting the mathematical model to experimental points, representing the decrease of MR in time of drying, with the highest possible values of determination coefficient (*R*^2^) and the lowest values of root mean square error (RMSE).

## 4. Conclusions

To the best of our knowledge, this is the first work comparing drying kinetics, hydroxycinnamic acids, carotenoids, color and sensory profile of ‘Algar’ loquat fruit under freeze-, convective-, vacuum-microwave- and combined-drying methods. On the basis of the results obtained connected with color, the freeze-drying technique maintained the most L* and b*, while vacuum-microwave drying showed the lowest. As expected, freeze drying ΔE was the lowest, similar to combined drying conditions, whilst the highest value was found after convective drying. The convective drying resulted in the highest hydroxycinnamic acids, the second-best results were obtained by using combined drying, followed by freeze drying. Regarding carotenoids, the highest values were obtained by the freeze-drying technique, followed by all convective treatments and vacuum-microwave at 360 W. The use of vacuum-microwaves significantly reduced the drying time, particularly at higher wattages. All treatments were sensory acceptable (no off-flavors) but with different characteristic flavor notes (especially loquat ID) and texture attributes. However, still, further research is needed to fully optimize these studied drying treatments because the freeze-dried sample still had higher carotenoid content and better instrumental color parameters. Therefore, it is worth continuing this research topic and studying the effect of other drying techniques, such as osmotic pre-treatment, on quality parameters, to obtain information about the biological activities of these novel products, to calculate the energy consumption and cost of the drying treatments and to obtain the international consumer acceptance drivers. Improving the quality of dried loquat might influence the popularity of this under-utilized fruit, even in groups with reduced fruit consumption, such as teenagers and children, resulting in higher product demand, and finally, higher benefits for European Union geographical indication schemes (Protected Designation of Origin, especially Nísperos Callosa d’En Sarrià) and the farmers and industry as well, protecting specific know-how, authenticity and agro-environmental conditions.

## Figures and Tables

**Table 1:** Impact of the drying techniques on parameters *A*, *k* and *n* of the modified Page’s model used to describe the drying kinetics of loquat fruit and MC (moisture content), a_w_ (water activity) and color coordinates (L*, a*, b* C*, H°, ΔE).

Treatment ^±^	Drying Kinetics $MR=A\cdot e^{-k\cdot \tau^{n}}$					RMSE ^‡^	*R* ^2^	MC (g/100 g ww)	a_w_	Color Coordinates ^¥^					
	*A*	*k*	*n*	τ											
				*CD*	VMD					L*	a*	b*	C*	H°*	*ΔE*
FD	- ^λ^	-	-	-	-	-	-	2.80 ± 0.09 ^c^	0.184 ± 0.001 ^c^	54.4 ± 0.3 ^a^	9.17 ± 0.12 ^ab^	20.9 ± 0.6 ^a^	22.8 ± 0.6 ^a^	66.3 ± 0.9 ^a^	7.1 ± 2.4 ^c^
CD 50	1	0.013	0.96	660	-	0.0049	0.9998	8.38 ± 0.07 ^a^	0.383 ± 0.007 ^a^	38.2 ± 2.3 ^b^	10.20 ± 1.27 ^a^	15.7 ± 3.3 ^ab^	18.7 ± 3.5 ^b^	56.7 ± 2.5 ^bc^	11.2 ± 3.5 ^b^
CD 60	1	0.011	1.04	540	-	0.0052	0.9997	6.76 ± 0.05 ^ab^	0.321 ± 0.001 ^ab^	41.0 ± 4.4 ^b^	12.10 ± 3.47 ^a^	18.5 ± 6.3 ^a^	22.3 ± 7.2 ^a^	56.5 ± 1.5 ^bc^	9.7 ± 3.4 ^c^
CD 70	1	0.013	1.08	420	-	0.0059	0.9997	5.19 ± 0.43 ^b^	0.282 ± 0.009 ^b^	36.7 ± 1.8 ^c^	8.02 ± 2.21 ^b^	13.1 ± 2.0 ^ab^	15.4 ± 2.9 ^c^	58.7 ± 3.1 ^b^	13.8 ± 2.7 ^b^
VMD 240	1	0.053	1.02	-	112	0.0089	0.9988	8.24 ± 0.29 ^a^	0.366 ± 0.030 ^a^	34.4 ± 2.5 ^d^	7.88 ± 2.03 ^b^	9.2 ± 3.1 ^b^	12.1 ± 3.7 ^d^	49.1 ± 2.3 ^d^	18.0 ± 4.0 ^a^
VMD 360	1	0.072	1.03	-	81	0.0142	0.9970	7.06 ± 0.58 ^a^	0.352 ± 0.070 ^a^	33.4 ± 0.7 ^d^	7.55 ± 0.91 ^b^	7.6 ± 0.5 ^c^	10.7 ± 1 ^e^	45.4 ± 1.4 ^d^	19.8 ± 0.9 ^a^
VMD 480	1	0.079	1.13	-	48	0.0098	0.9987	7.49 ± 0.09 ^a^	0.336 ± 0.012 ^a^	36.0 ± 1.3 ^c^	8.30 ± 0.39 ^b^	10.8 ± 2.1 ^b^	13.7 ± 1.9 ^cd^	52.2 ± 4.1 ^c^	15.6 ± 2.3 ^a^
CPD-VMFD	0.086	0.259	0.729	180	30	0.0046	0.9595	6.39 ± 0.23 ^ab^	0.303 ± 0.016 ^ab^	39.7 ± 0.1 ^b^	8.94 ± 0.95 ^ab^	15.7 ± 0.2 ^ab^	18.0 ± 0.7 ^b^	60.3 ± 2.3 ^b^	9.7 ± 0.9 ^c^

**^±^** Freeze drying (FD), convective drying (CD) at 50 °C (CD 50), 60 °C (CD 60) and 70 °C (CD 70); vacuum-microwave drying (VMD) at 240 W (VMD 240), 360 W (VMD 360) and 480 W (VMD 480) and combined drying (CPD-VMFD). ^¥^ Mean values followed by different letter, within the same column, were significantly different (*p* < 0.05). ***^‡^*** RMSE: root mean square error.; ^λ^ “-“ means no data available of this parameters.

**Table 2:** Characteristic bioactive compounds of dried loquat.

Code	t_R_ (min)	λ_max_ (nm)	MS (*m/z*)//MS/MS (*m/z*) ^¥^	Compounds
Hydroxycinnamic acid derivatives (H)				
3-CQA	3.04	321	359//191/179/135	3-caffeoylquinic acid
3-*p*-CoQA	3.75	310	337//191/173/163	3-*p*-coumaroyl quinic acid
5-CAQ	3.98	321	337//191/173/163	5-caffeoylquinic acid
SG	4.20	299	385//162/223	sinapoyl glucoside
5-*p*-CoQA	4.67	299	337//173	5-*p*-coumaroylquinic acid
5-FQA	5.53	324	367//193/191	5-feruloylquinic acid
Carotenoids (C)				
Zea	7.25	450/480	569//551/533/476	Zeaxanthin
β-C	8.52	454/481	536//444	β-Carotene
*cis-*β-C	8.71	450/474	536//444	15,15-*cis-*β-Carotene
β-Cp	10.13	446/479	791//698/535/443	β-Cryptoxanthin–palmitate (C16:0)
Neox	11.00	447.475	601//583/565/393	Neoxanthin
Zeam	11.97	455/478	807//551	Zeaxanthin monopalmitate
β-Cpm	12.13	451/482	791	β-Cryptoxanthin monopalmitate

^¥^ MS: mass spectrometry; MS/MS: tandem mass spectrometry.

**Table 3:** Effect on hydroxycinnamic acid derivatives, carotenoids compounds and antioxidant capacity ABTS^+•^ and FRAP radical of dried loquat (ww).

Sample ^±^	Hydroxycinnamic Acid Derivatives (H) *^†^*^¥^							Carotenoids (C) *^††^*^¥^								Antioxidant Capacity ^¥^	
	3-CQA	3-*p*-CoQA	5-CAQ	SG	5-*p*-CoQA	5-FQA	∑H	Zea	β-C	*cis*-β-C	β-Cp	Neox	Zeam	β-Cpm	∑C	ABTS^+•^	FRAP
	mg/kg ww							mg/kg ww								mmol Trolox/100 g ww	
FD	1282 ± 23 ^b^	274.2 ± 12.5 ^b^	2548 ± 23 ^b^	151.0 ± 10.3 ^a^	17.2 ± 1.2 ^b^	170.7 ± 1.2 ^a^	4394 ^c^	29.32 ± 2.55 ^b^	1182 ± 12 ^a^	83.55 ± 3.71 ^b^	445.5 ± 32.1 ^a^	189.0 ± 3.5 ^a^	225.6 ± 23.1 ^a^	446.4 ± 13.2 ^a^	2601 ^a^	3.08 ± 0.43 ^b^	2.21 ± 0.19 ^b^
CD 50 °C	1411 ± 12 ^a^	269.2 ± 13.4 ^b^	2913 ± 34 ^a^	114.9 ± 9.5 ^b^	8.41 ± 0.14 ^c^	165.2 ± 2.6 ^a^	4882 ^ab^	43.02 ± 3.66 ^a^	575.4 ± 21.3 ^b^	59.19 ± 2.43 ^c^	220.3 ± 12.5 ^b^	97.4 ± 5.3 ^b^	85.13 ± 10.31 ^b^	193.9 ± 8.3 ^b^	1274 ^b^	2.88 ± 0.27 ^bc^	1.54 ± 0.21 ^c^
CD 60 °C	1474 ± 21 ^a^	268.4 ± 10.8 ^b^	3055 ± 21 ^a^	163.7 ± 7.4 ^a^	9.21 ± 0.77 ^c^	168.5 ± 3.1 ^a^	5139 ^a^	41.55 ± 3.71 ^a^	520.3 ± 2.8 ^bc^	53.30 ± 3.65 ^c^	185.5 ± 10.4 ^c^	80.39 ± 4.7 ^b^	76.86 ± 9.43 ^b^	178.7 ± 2.54 ^bc^	1136 ^b^	3.14 ± 0.11 ^ab^	1.86 ± 0.10 ^c^
CD 70 °C	1455 ± 22 ^a^	289.4 ± 9.5 ^b^	3116 ± 18 ^a^	157.7 ± 10.7 ^a^	6.36 ± 8.51 ^c^	187.9 ± 4.8 ^a^	5211 ^a^	37.89 ± 3.21 ^a^	448.4 ± 8.6 ^c^	117.0 ± 11.8 ^a^	175.8 ± 10.6 ^c^	73.72 ± 5.32 ^b^	81.90 ± 9.11 ^b^	193.4 ± 2.7 ^b^	1128 ^b^	3.80 ± 0.54 ^a^	2.26 ± 0.09 ^b^
VMD 240 W	982.0 ± 10.6 ^c^	258.0 ± 18.4 ^b^	1751 ± 20 ^d^	128.4 ± 11.7 ^ab^	24.3 ± 2.1 ^a^	152.5 ± 1.9 ^b^	3297 ^d^	16.02 ± 2.61 ^b^	364.1 ± 2.6 ^d^	10.91 ± 0.99 ^d^	179.4 ± 13.5 ^c^	78.25 ± 5.78 ^b^	68.93 ± 7.91 ^b^	151.4 ± 5.3 ^c^	869.0 ^d^	2.12 ± 0.88 ^c^	1.77 ± 0.12 ^c^
VMD 360 W	1273 ± 25 ^b^	352.0 ± 25.4 ^a^	2374 ± 16 ^b^	168.3 ± 12.6 ^a^	19.2 ± 1.9 ^b^	149.1 ± 5.6 ^b^	4336 ^c^	19.74 ± 1.34 ^c^	612.3 ± 7.5 ^b^	53.68 ± 1.23 ^c^	217.4 ± 23.5 ^b^	107.1 ± 2.5 ^b^	83.31 ± 2.55 ^b^	214.1 ± 7.2 ^b^	1308 ^b^	0.97 ± 0.07 ^d^	0.97 ± 0.04 ^d^
VMD 480 W	1059 ± 27 ^bc^	243.7 ± 25.3 ^b^	2499 ± 18 ^b^	158.1 ± 10.4 ^a^	22.0 ± 1.5 ^a^	126.7 ± 6.3 ^b^	4109 ^c^	16.25 ± 1.32 ^c^	408.8 ± 12.5 ^d^	69.08 ± 3.67 ^bc^	167.0 ± 12.4 ^c^	87.90 ± 7.31 ^b^	92.06 ± 4.65 ^b^	201.5 ± 9.1 ^b^	1043 ^c^	3.02 ± 0.51 ^b^	3.11 ± 0.11 ^a^
CPD-VMFD	1378 ± 15 ^b^	267.1 ± 11.0 ^b^	2621 ± 16 ^b^	199.7 ± 9.3 ^a^	23.0 ± 1.4 ^a^	162.7 ± 7.7 ^a^	4651 ^b^	30.29 ± 2.76 ^b^	455.2 ± 9.8 ^c^	55.27 ± 6.32 ^c^	181.8 ± 4.8 ^c^	78.18 ± 8.54 ^b^	92.16 ± 12.5 ^b^	197.5 ± 10.9 ^b^	1090 ^c^	2.23 ± 0.37 ^c^	2.29 ± 0.23 ^b^

***^†^*** 3-CQA: 3-caffeoyl quinic acid; 3-*p*-CoQA: 3-*p*-coumaroyl quinic acid; 5-CAQ: 5-caffeoyl quinic acid; SG: sinapoylglucoside; 5-*p*-CoQA: 5-*p*-coumaroyl quinic acid; 5-FQA: 5-feruloylquinicacid; ∑H: sum of hydroxycinnamic acids ***^††^*** Zea: Zeaxanthin; β-C: β-carotene; *cis*-β-C: 15,15-*cis*-β-carotene; β-Cp: β-cryptoxanthin palmitate; Neox: neoxanthin; Zeam: zeaxanthin monopalmitate; β-Cpm: β-cryptoxanthin monopalmitate; ∑C: sum of carotenoids; **^±^** FD: Freeze drying; CD: convective drying at 50 °C (CD 50), 60 °C (CD 60) and 70 °C (CD 70); VMD: vacuum-microwave drying at 240 W (VMD 240), 360 W (VMD 360) and 480 W (VMD 480); CPD-VMFD: combined drying. ^¥^ Mean values followed by different letter, within the same column, were significantly different (*p* < 0.05).
